# Research on strategies for enhancing the competency of safety directors in EPC projects empowered by AI

**DOI:** 10.1371/journal.pone.0348868

**Published:** 2026-05-18

**Authors:** Jing Guan, Zhen chao Yang, Congcong Wang

**Affiliations:** 1 College of Economics and Management, Beijing Jiaotong University, Beijing, China; 2 Shaanxi Construction Engineering No.8 Construction Group Co., Ltd., Xi’an, Shaanxi, China; 3 General Contracting Company of China Construction Third Engineering Bureau Co., Ltd., Wuhan, Hubei, China; University of Karachi, PAKISTAN

## Abstract

With the widespread application of the engineering–procurement–construction (EPC) delivery model in large-scale infrastructure and complex industrial projects, the highly dynamic construction environment and the escalating complexity of risks pose heightened requirements for the competency of project safety directors. Conventional approaches that rely primarily on experience and manual inspections have become a bottleneck to further improvements in safety management capability. This paper investigates strategies to enhance the competency of safety directors in EPC projects through AI empowerment. Drawing on dynamic capability theory, synergy theory, and the Technology Acceptance Model (TAM), we develop a competency enhancement framework comprising three dimensions—sensing, seizing, and reconfiguring. A comparative experimental design and the Analytic Hierarchy Process (AHP) were employed for empirical evaluation. The results indicated that the Comprehensive Competency Index (CCI) under the AI-empowered mode increased by 45.9% relative to the traditional mode, enabling a shift in safety management from experience-driven practices to data- and intelligence-driven governance. Furthermore, this study proposes a tiered improvement strategy and a quantitative evaluation system, offering theoretical grounding and practical guidance for talent selection and digital-intelligent transformation in construction enterprises..

## 1. Introduction

Owing to its outstanding performance in resource integration, risk sharing, and life-cycle optimization, the EPC model has become a dominant project delivery paradigm for large-scale infrastructure and complex industrial projects worldwide [1,2]. In EPC projects, the high degree of coupling among design, procurement, and construction activities creates multiple challenges at construction sites, including numerous dynamic risk sources, complex stakeholder coordination, and harsh working conditions [3,4]. As a cornerstone of project implementation, safety management performance is directly shaped by the personal qualities and decision-making effectiveness of the safety director [5,6]. The safety director serves not only as the maker of safety policies but also as the central leader in risk control and emergency response; accordingly, his or her competency constitutes a key determinant of project safety performance [7,8]. However, traditional safety management paradigms rely excessively on individual experience and manual inspections, and therefore often suffer from delayed perception, subjective decision-making, and insufficient control coverage when confronted with massive heterogeneous data and sudden compound risks [9,10].

Before examining the competency of safety directors, it is essential to clarify the core characteristics of EPC projects and the specific challenges they pose to safety management. Unlike the traditional construction general contracting model, EPC projects are typically characterized by the high degree of interdependence among design, procurement, and construction; the involvement of numerous cross-disciplinary stakeholders; and the dynamic evolution of risks throughout the entire project life cycle. In such highly complex and dynamic operating environments, on-site safety information is often marked by multi-source heterogeneity and explosive data growth. Traditional safety management approaches rely heavily on the personal experience of safety directors and manual site inspections. When confronted with EPC-specific information barriers, such as the disconnection between design and construction, as well as sudden compound risks, these approaches are prone to delayed perception and reactive response. Therefore, in the EPC context, leveraging advanced technologies to overcome the limits of individual cognitive bandwidth and enhance the competency of safety directors in addressing complex system risks is not only crucial to successful project delivery but also an urgent imperative for the transformation of the engineering management paradigm.

In recent years, digital-intelligent technologies represented by AI have rapidly penetrated the engineering construction sector, driving a paradigm shift in safety management from post-incident response and in-process control to pre-incident prediction and real-time intervention [11]. The integrated application of technologies such as Computer Vision, Deep Learning, Natural Language Processing, and Digital Twin has provided a traceable and iterative technological foundation for safety management in EPC projects [12,13]. In the area of visual recognition and on-site perception, Kulinan et al. [14] proposed a real-time monitoring method for construction workers’ safety hazards by integrating BIM and computer vision, enabling localization and risk identification based on BIM semantics and on-site visual data, with an average localization error of only 13.2 cm. Xu et al. [15] developed a real-time abnormal behavior recognition network for factory personnel by incorporating dual-layer routing attention and efficient convolution modules, thereby achieving accurate real-time identification of multiple categories of abnormal worker behavior. Wu et al. [16] integrated digital twin, deep learning, and mixed reality technologies to establish a real-time visual early warning system for construction sites, which enabled the real-time presentation of hazard information and proactive risk avoidance. In terms of multi-source data fusion and predictive warning, Wang et al. [17] proposed MGTCN, a multi-graph spatiotemporal forecasting model combining GNN and TCN, and integrated it with a multi-source data fusion and multi-graph construction framework to achieve multi-step risk prediction with high accuracy and strong robustness. Sun et al. [18] designed a dynamic risk early warning system that integrated knowledge-driven consequence data with data-driven likelihood data; validation results showed that the system could accurately characterize uncertainty and deliver rapid dynamic warnings. In the area of text mining and knowledge modeling, Isah et al. [19] constructed a knowledge graph for construction safety risks and, by integrating information on accident cases, hazard sources, and control measures, enabled risk prevention, control, and decision support. Zhang et al. [20] proposed a BiLSTM model that incorporated TextRank-Word2vec fuzzy word vectors and a self-attention mechanism to automatically identify hazard information and assess risk severity, achieving an accuracy of 91.70% and effectively improving construction safety management efficiency. Regarding virtual-real mapping and system simulation, Moe et al. [21] proposed a digital twin framework for safety monitoring based on Federated Learning, enabling real-time safety prediction and monitoring at construction sites under resource-constrained conditions. Shi et al. [22] incorporated spatiotemporal information into a digital twin model to achieve coordinated control of point cloud optimization, inspection, and real-time monitoring, reducing error detection time by 30% and keeping construction errors within 3%. Han et al. [23] developed a digital twin management framework for construction safety from the 4M1E perspective and, based on a review of 113 publications, summarized the research progress, gaps, and challenges, while proposing future research directions at the digital, model, and application layers to support the intelligent upgrading of construction safety management. Collectively, these studies have expanded the technological boundaries of safety management in EPC projects across four dimensions—perception, prediction, knowledge representation, and simulation—and have promoted a shift in safety management logic from experience-driven approaches to data-driven and model-driven ones.

However, improvements in algorithmic performance do not necessarily translate into better safety management outcomes. The TAM proposed by Davis et al. [24] demonstrated that perceived usefulness and perceived ease of use significantly influence individuals’ adoption of new technologies, thereby providing a classical framework for explaining whether AI systems are embraced or rejected in safety management contexts. Zhu et al. [25] further incorporated social influence and facilitating conditions into the UTAUT model, highlighting the decisive role of organizational support and the institutional environment in successful technology implementation. In the area of safety-related human–machine collaboration, Zhao et al. [26] proposed an intelligent method for constructing an index system for offshore wind farm site selection driven by human–machine collaboration; the results showed that such collaboration significantly improved the scientific rigor and effectiveness of standard construction for offshore wind farm siting. To assess trust risk in human–machine collaboration scenarios, Wang et al. [27] developed a dynamic evaluation method integrating an improved cloud model with a Bayesian network, enabling trust risk prediction, identification of key factors, and targeted intervention, thereby effectively enhancing the safety and efficiency of human–machine collaboration. From the perspective of organizational capability, Teece et al. [28] proposed dynamic capability theory, emphasizing that organizations achieve competitive advantage in rapidly changing environments through sensing, seizing, and reconfiguring. This perspective provides a theoretical foundation for explaining how safety directors reconstruct governance capability during digital-intelligent transformation. Taken together, these studies suggest that, in an AI-enabled context, the competency of safety directors should extend beyond engineering safety knowledge and on-site management skills to include composite dimensions such as data literacy, model understanding, human–machine collaboration, and ethical governance. Moreover, such competency enhancement needs to be supported by institutionalized process reengineering and closed-loop feedback mechanisms so as to realize synergistic gains across technology, humans, and organizations.

In summary, the existing literature has largely focused on optimizing the performance of technological tools, while rarely examining, from a management perspective, how AI reshapes the competency structure and cognitive boundaries of project safety directors in a systematic manner. To address this gap, this paper integrates dynamic capability theory with the TAM to develop a “sensing–seizing–reconfiguring” competency enhancement model for safety directors in EPC projects under AI-enabled conditions. A long-term comparative experiment was conducted to quantitatively verify the significant effect of AI-enabled approaches on improving the Comprehensive Competency Index (CCI). On this basis, the study further proposes an operational tiered enhancement strategy, thereby providing theoretical support and practical guidance for talent evaluation and organizational upgrading in construction enterprises during digital-intelligent transformation.

## 2. Theory and methodology

### 2.1 Theoretical background

In the safety management of EPC projects, the competency of the project safety director is a critical factor in ensuring construction safety and project quality [5,6]. With the rapid development of AI technologies in engineering management, the competency structure and management mode of this role are undergoing profound transformation [29]. To explain this transformation, this paper integrates Dynamic Capability Theory, Human-AI Collaboration Theory, and the TAM, thereby providing systematic theoretical support for the AI-enabled competency enhancement mechanism of safety directors in EPC projects from multiple dimensions, including individual competency composition, organizational adaptability, human–AI interaction mechanisms, and technology adoption behavior.

#### 2.1.1 Dynamic capabilities theory.

Dynamic Capability Theory, proposed by Teece, is used to explain how organizations maintain competitive advantage in uncertain environments [28]. The theory centers on three core capabilities: sensing, seizing, and transforming. In the context of EPC safety management, sensing capability is reflected in the safety director’s ability to identify potential risks through AI-based analysis, seizing capability is reflected in the rapid formulation and implementation of risk response strategies, and transforming capability is manifested in the continuous optimization of safety processes and safety culture. AI technologies strengthen all three capabilities. Particularly in complex environments characterized by multiple projects operating in parallel, Dynamic Capability Theory provides an effective framework for understanding the competency enhancement of safety directors.

#### 2.1.2 Human-AI collaboration theory.

Human–AI Collaboration Theory emphasizes task allocation and trust building between humans and automated systems [30]. It suggests that automation should capitalize on its strengths in information processing, pattern recognition, and rapid data analysis, while humans remain indispensable in value judgment and the interpretation of complex situations. In the context of EPC safety management, AI systems can undertake functions such as real-time monitoring and data analysis, whereas safety directors retain a leading role in contextual understanding and ethical decision-making. Trust is a critical factor in effective human–AI collaboration: insufficient trust may lead to the disregard of system recommendations, while excessive trust may result in serious consequences when the system fails or produces erroneous outputs.

#### 2.1.3 Technology acceptance model.

The Technology Acceptance Model, proposed by Davis, suggests that individuals’ intention to adopt a technology is primarily influenced by perceived usefulness and perceived ease of use [24]. In the context of EPC safety management, if safety directors perceive that AI can significantly improve management efficiency and is easy to use, they are more likely to actively adopt the technology. Conversely, complex or user-unfriendly system design may reduce adoption rates and weaken the effectiveness of AI enablement. Therefore, TAM provides an important theoretical basis for analyzing the psychological mechanisms underlying safety directors’ adoption of AI.

### 2.2 Methodology

#### 2.2.1 Comparative experimental method.

To scientifically quantify the empowering effect of AI technology on the competency of safety directors in EPC projects, this study adopts a classical quasi-experimental design widely used in engineering management and organizational behavior research [31]. By establishing an experimental intervention and a control baseline, this approach makes it possible to effectively isolate the impact of the key independent variable—AI enablement—on competency outcomes. The experimental procedure follows the standard sequence of pre-test baseline calibration, intervention implementation, and post-test effect evaluation.

In addition, to minimize the potential interference of differences in participants’ backgrounds, a homogeneity test was conducted prior to random grouping. This ensured that any subsequent differences in the competencies demonstrated by project safety directors in core tasks such as risk control, collaborative management, and strategic planning could be more clearly attributed to the AI-enabled intervention.

#### 2.2.2 Analytic hierarchy process.

Since the evaluation of project safety directors’ competency involves multiple complex and interrelated dimensions, this study employs the AHP to construct a scientific weighting system. By decomposing a complex decision problem into a target layer, a criterion layer, and an indicator layer, AHP can effectively reduce the influence of subjective bias in competency evaluation [32]. Based on the three-dimensional framework of “sensing–seizing–reconfiguring” derived from Dynamic Capability Theory, this study develops a hierarchical structural model and convenes an expert committee composed of senior specialists, scholars, and industry regulators in the field of EPC safety management.

During the implementation process, the Saaty 1–9 scale was employed for pairwise comparisons to construct the group judgment matrices. To ensure the logical rigor of the weight allocation process, the geometric mean method was adopted to aggregate expert judgments, and strict consistency tests were conducted. A consistency ratio *CR* of less than 0.1 was used as the criterion for determining that the judgment matrices exhibited acceptable logical consistency.

## 3. AI-enabled competency enhancement model

### 3.1 Model design philosophy

With the growing penetration of AI technology into the field of construction safety, project safety directors are gradually shifting from traditional risk supervisors, whose work primarily relied on manual oversight, to technology-driven safety architects. This transformation is manifested not only in changes to their job content, but also in the restructuring of their competency profile.

Drawing on Human–AI Collaboration Theory and Dynamic Capability Theory, this study proposes a dual role for AI in shaping the competency of EPC project safety directors. On the one hand, AI functions as a “cognitive prosthesis”, extending safety directors’ sensing and cognitive capabilities. On the other hand, it serves as an “efficiency multiplier”, reducing the time required for information processing and decision-making, thereby freeing cognitive and temporal resources for higher-level strategic thinking.

The concept of a “cognitive prosthesis” originates from research in cognitive science and assistive technology. At its core, it refers to the use of external technological tools to compensate for or extend the boundaries of human perception and cognition. In complex EPC project environments, on-site safety information is often characterized by multi-source heterogeneity, real-time dynamics, and massive scale, far exceeding what safety directors can effectively process through experience and intuition alone. The introduction of AI makes it possible for safety directors to leverage technologies such as Computer Vision, Natural Language Processing (NLP), and Machine Learning (ML) to complete information acquisition, risk identification, and decision analysis within seconds. These capabilities allow safety directors to transcend the natural physiological and cognitive limits of human information processing in both the depth and breadth of their understanding of construction-site data, thereby producing a qualitative leap in the “sensing” dimension.

In contrast, the notion of an “efficiency multiplier” highlights the role of AI in information processing and decision execution. In traditional safety management processes, the progression from data collection and organization to analysis and decision-making often requires hours or even days. This not only delays the optimal window for safety intervention, but also forces safety directors to devote substantial effort to low-value-added technical operations. Through automated data processing, real-time analysis, and visualized presentation, AI systems can compress this process to minutes or even seconds. Such dramatic gains in efficiency enable safety directors to redirect more time and attention toward strategic safety planning and cross-departmental coordination, thereby enhancing their capacity to convert opportunities into effective action in the “seizing” dimension.

Building on the above theoretical foundations and the dual role positioning of AI, this study defines the AI empowerment pathway as a progressive three-level process for systematically enhancing the competency of project safety directors. Specifically, the pathway takes the “sensing–seizing–reconfiguring” framework of Dynamic Capability Theory as its core logic, the optimal task allocation mechanism of Human–AI Collaboration Theory as its operational mechanism, and the enhancement of adoption intention emphasized in the Technology Acceptance Model as its enabling condition. Through the deep embedding of AI into the full lifecycle of EPC project safety management, this pathway realizes the systematic empowerment of project safety directors’ competency. Specifically, it includes:

The first level is the sensing-level empowerment pathway, which centers on AI-enabled multi-source data fusion, computer vision, and risk identification algorithms. This pathway breaks through the physiological limits of safety directors’ perception and the constraints of experience-based cognition, thereby expanding the radius of risk perception and improving the comprehensiveness, accuracy, and timeliness of risk identification. In doing so, it enables a leap in cognitive capability from ex post response to ex ante prediction.

The second level is the seizing-level empowerment pathway, which centers on AI-driven intelligent decision-making models, hierarchical early-warning mechanisms, and collaborative management platforms. This pathway compresses the time required for information processing and decision circulation, optimizes risk response processes and cross-actor coordination mechanisms, and enhances the scientificity of safety decision-making, the efficiency of response actions, and the smoothness of collaboration. In doing so, it enables a leap in managerial effectiveness from a transactional executor to a strategic controller.

The third level is the reconfiguring-level empowerment pathway, which centers on AI-enabled personalized training systems, knowledge management systems, and full-lifecycle digital twin simulation. This pathway reshapes the organizational processes, learning systems, and cultural foundations of safety management, while enhancing safety directors’ capabilities for continuous learning, strategic planning, and regulatory adaptation. In doing so, it enables a leap in systemic capability from experience-driven management to digital-intelligent governance.

In summary, the competency enhancement model developed in this study is centered on the three dimensions of sensing, seizing, and reconfiguring. By integrating the task-allocation logic of Human–AI Collaboration Theory, the model facilitates the organic fusion of AI technologies and human capabilities, thereby advancing the competency enhancement of project safety directors.

### 3.2 Technical composition of the AI-enabled environment

To systematically examine the empowering effect of AI technologies on the competency of safety directors in EPC projects, this study conceptualizes AI as the foundational support for enabling safety directors to develop the dynamic capabilities of sensing, seizing, and reconfiguring. The AI-enabled environment employed in the experiment consists of three interrelated categories of core technologies:

1. Multi-source sensing technologies. This category primarily comprises computer vision and edge computing. Through the deployment of on-site video surveillance systems and the application of object detection algorithms, it enables the real-time capture of unsafe worker behaviors, such as non-compliant operations and inadequate protective measures, as well as unsafe material conditions. As a result, the perceptual scope of safety directors is extended from limited manual inspection to continuous and comprehensive digital monitoring, effectively mitigating the perception lag caused by the complexity of EPC construction environments.
2. Predictive decision-making technologies. This category is centered on ML and big data analytics. By exploring the correlations among historical hazard records, meteorological information, and schedule data, the platform develops risk early-warning models. This allows safety directors to capture latent risk coupling relationships that are difficult to identify through direct observation, while enabling the system to automatically generate early warnings and preliminary response recommendations. Consequently, it significantly improves both the efficiency of risk capture and the scientificity of early-warning decision-making in complex environments.
3. Knowledge reconfiguration and collaborative technologies. This category mainly involves NLP and DW technology. NLP performs semantic parsing of large volumes of industry regulations, accident cases, and project logs, thereby assisting safety directors in rapidly building knowledge graphs. Digital twin technology further integrates the outputs of AI analysis into the BIM model for visual representation. This provides an intuitive digital interface for reconfiguring project safety management processes and facilitating cross-actor collaboration, thereby promoting the transformation of safety management from experience-driven practices to digitally intelligent governance.

### 3.3 Core model architecture

To systematically depict the process of competency enhancement among safety directors under AI empowerment, this study develops a variable-system equation. Specifically, the input variables consist of AI technological capability ($A_{c}$), the individual capability of the safety director ($H_{c}$), and organizational resource investment ($O_{r}$). The process variables correspond to the three dimensions of dynamic capability, namely AI-enabled risk sensing ($A_{s}$), AI-assisted decision seizing ($A_{e}$), and AI-supported organizational reconfiguration ($A_{t}$). The output variables include safety performance ($S_{p}$) and the competency improvement index ($C_{i}$). The variable-system equation of the proposed model is expressed as follows:


$$\begin{array}{c} S_{q}=\alpha_{\text{1}}A_{s}+\alpha_{\text{2}}A_{e}+\gamma_{\text{1}}A_{t} \end{array}$$ (1)

$$\begin{array}{c} C_{i}=\beta_{\text{1}}A_{s}+\beta_{\text{2}}A_{e}+\beta_{\text{3}}A_{t} \end{array}$$ (2)

**Fig 1:**
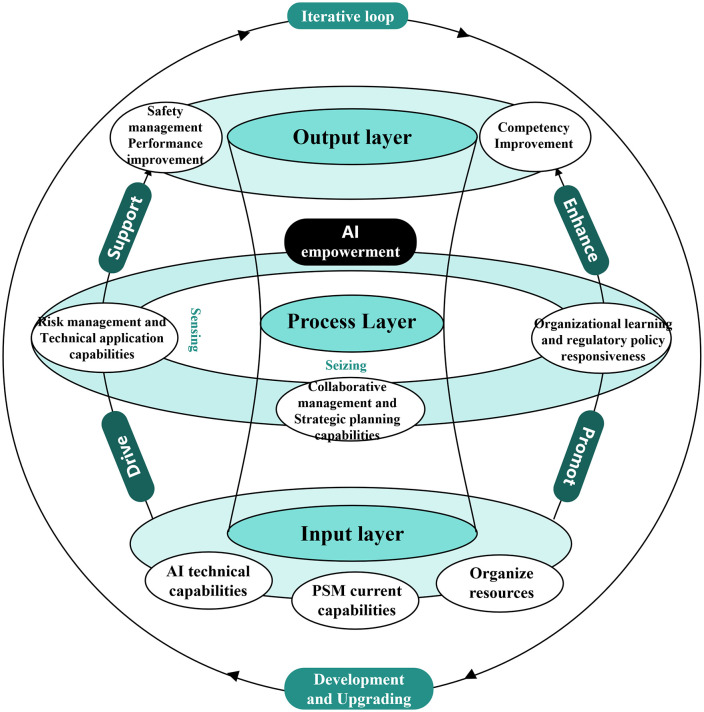
Competency enhancement model.

The above equation indicates that, under the support of AI technological capability, the sensing and seizing dimensions primarily affect safety performance, whereas the reconfiguring dimension not only enhances safety performance but also plays a significant role in improving competency. Reconfiguring capability does not merely optimize existing systems and mechanisms; it also reshapes the cognitive modes of both organizations and individuals in safety management, thereby fostering higher-level strategic orientation and leadership style. Based on this equation, the competency enhancement model developed in this study is presented in Fig 1.

As illustrated in Fig 1, the proposed model is organized into a three-layer framework. The input layer includes AI technological capability, the capability of safety directors, and organizational resources. The process layer reflects the three dimensions of dynamic capability “sensing, seizing, and reconfiguring” which are further operationalized as six core competency dimensions of safety directors under AI empowerment: risk control, technology application, collaborative management, strategic planning, continuous learning, and regulatory and policy tracking. The output layer consists of safety performance and competency enhancement. Meanwhile, the model also incorporates a feedback loop, whereby improvements in the output layer in turn influence the input layer. For instance, enhanced safety performance may motivate organizations to increase investment in AI technologies and organizational resources, thereby further strengthening the capability of safety directors in the subsequent cycle.

In summary, the AI-enabled competency enhancement model proposed in this chapter explains the underlying mechanism through which AI technologies deeply empower the competency of safety directors in EPC project safety management. The model not only incorporates a complete “input, process, output” chain at the structural level, but also possesses sustainable optimization potential through its closed-loop feedback mechanism.

## 4. Analysis of competency enhancement under AI enablement

### 4.1 Enhancing front-end risk control and technology application capabilities

Risk control capability and technology application capability are the core competencies underpinning front-end safety management by safety directors, and they also represent the major capability shortcomings in traditional management models. Conventional risk control primarily depends on the managerial experience of safety directors, and therefore suffers from limitations such as restricted perceptual scope, high rates of missed judgment, and delayed response. In contrast, by constructing a closed-loop risk management system integrating “multi-source data fusion, AI-based model analysis, and hierarchical early warning and response”, AI technologies can serve as a form of “cognitive prosthesis” for safety directors, enabling them to transcend physiological and experiential constraints and thereby upgrade risk management from passive reaction to proactive anticipation [33].

#### 4.1.1 Multi-source data fusion.

Multi-source data fusion technology provides an important foundation for improving the risk identification capability of safety directors, and its essential role lies in expanding the “radius of risk perception”. Through the deployment of an integrated data acquisition network, multidimensional data concerning personnel, equipment, environmental conditions, and management activities at the construction site can be captured and standardized in real time. The effectiveness of this network is directly related to the risk identification capability of safety directors, and its quantitative indicator can be expressed by the following equation:


$$\begin{array}{c} C_{r}=0.5\frac{N_{\text{ac}}}{N_{\text{al}}}+0.3\frac{N_{\text{hi}}}{N_{\text{ha}}}+0.2\frac{T_{\text{ave}}}{T_{\text{sta}}} \end{array}$$ (3)

Where, *C*_r_ represents the risk identification capability score; *C*_r_ represents the actual number of hazards identified; *N*_al_ represents the total number of hazards confirmed in the same period through third-party inspection; *N*_hi_ represents the number of concealed hazards identified; *N*_ha_ represents the total number of concealed hazards confirmed in the same period; *T*_ave_ represents the average time between hazard occurrence and detection; and *T*_sta_ represents the standard detection time for hazards specified by the industry or the enterprise.

To examine the AI-enabled effect of the network, this study conducted a controlled experiment involving 20 project safety directors, each with more than 10 years of relevant professional experience. The participants were randomly assigned to an experimental group and a control group, with 10 individuals in each group. To minimize the potential influence of individual differences on subsequent evaluation outcomes, baseline homogeneity tests were conducted before random assignment, focusing on background knowledge and innovative technology capability. Background knowledge was represented by years of professional experience (with average values of 11.2 and 10.8 years for the two groups, respectively) and highest educational attainment. Innovative technology capability was assessed based on the TAM, using a pre-test questionnaire to quantify participants’ initial perceived usefulness and perceived ease of use of digital management tools. Independent-samples t-test results indicated no statistically significant differences between the experimental and control groups in either background knowledge (*t* = 0.42*, p* > 0.05) or initial acceptance of innovative technologies (*t* = −0.58, *p* > 0.05). This sugges*t*s that the two groups were highly homogeneous at the outset of the experiment, thereby establishing a reliable data basis for quantifying the net effect of AI enablement in the subsequent analysis.

**Fig 2:**
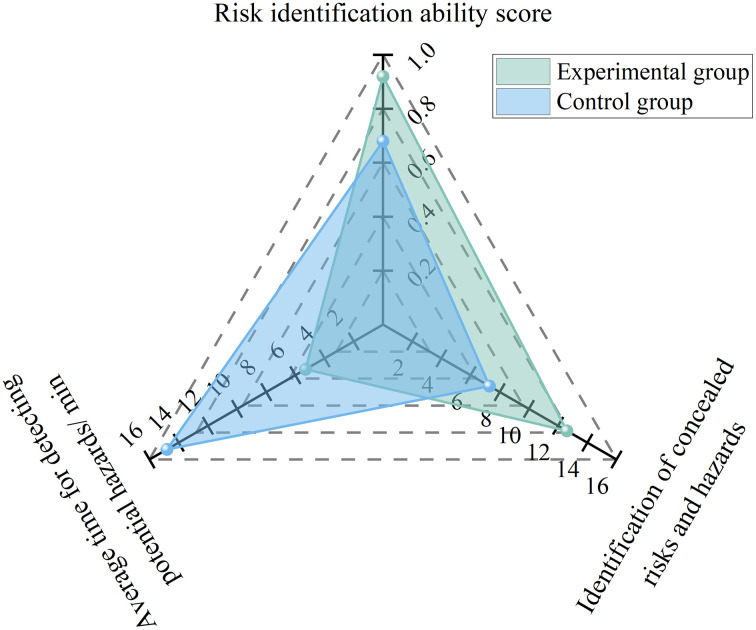
Experimental results for risk identification capability under AI enablement.

In the experiment, the 10 participants in the experimental group employed the AI-based data acquisition network, whereas the 10 participants in the control group continued to adopt the traditional manual collection approach. The six-month empirical results showed that the risk identification capability score of project safety directors in the experimental group increased from 0.68 to 0.92, representing a 35.3% improvement. Specifically, the number of concealed hazards identified increased from 7.3 to 12.6 cases per month, while the average time required for hazard detection decreased from 14.8 h to 5.3 h. The results are presented in Fig 2. These findings indicate that, under AI enablement, improvements in the accuracy and timeliness of data collection can be directly translated into greater comprehensiveness and precision in the risk identification process of safety directors, thereby effectively overcoming the limitations of the traditional mode, which is constrained by experience dependence and restricted perceptual scope.

#### 4.1.2 AI model application.

The deep application of AI models constitutes the core vehicle for improving safety directors’ capabilities in risk prediction, response, and technology application. All quantitative indicators are based on traceable data, including on-site records and tool operation logs, so as to ensure the field adaptability and empirical feasibility of model deployment. Since risks at EPC project sites are marked by dynamic evolution and coupling relationships, precise control can hardly be achieved through experience-based judgment alone. Through in-depth mining of massive on-site data and the extraction of latent patterns, intelligent models provide scientific decision support for safety directors and facilitate the transformation of risk management from experience-driven decision-making to data-driven governance.

(1)Risk Anticipation Capability

Risk prediction capability is centered on “The proportion of hazards that are warned of and rectified in advance”. Its core lies in leveraging AI models to mine the evolutionary patterns of risks, thereby enabling the proactive identification and mitigation of hazards. The quantitative calculation formula is as follows:


$$\begin{array}{c} S_{\text{w}}=0.6\frac{N_{\text{wa}}}{N_{\text{aw}}}+0.4\frac{T_{\text{ad}}}{T_{\text{bu}}} \end{array}$$ (4)

Where, *S*_w_ represents the risk prediction capability score; *N*_wa_ represents the number of risks rectified following advance warning; *N*_aw_ represents the total number of risk warnings issued; *T*_ad_ represents the lead time of the warning; and *T*_bu_ represents the buffer time available for rectification.

**Fig 3:**
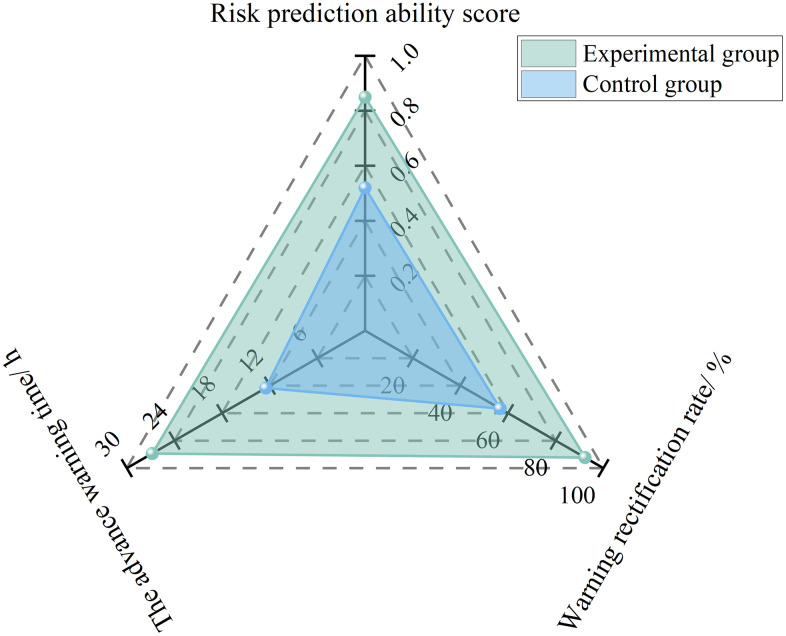
Experimental results for risk prediction capability under AI Enablement.

To evaluate the effect of AI models on improving safety directors’ risk prediction capability, 10 project safety directors were randomly selected from the experimental subjects described in Section 4.1.1 to conduct a six-month comparative experiment focusing on the prediction of safety risks associated with large-scale equipment. During the first three months, no AI model was used, while during the subsequent three months, AI models were introduced. As shown in Fig 3, after the application of AI models, the warning-based rectification rate increased from 56.7% to 92.3%, the advance warning time extended from 12.5 h to 26.8 h, and the risk prediction capability score rose from 0.52 to 0.85, indicating a substantial enhancement in the proactiveness of risk control. Furthermore, for high-risk equipment, such as tower cranes and shield machines, AI models are capable of identifying latent fault hazards that are difficult to detect through manual inspection alone by analyzing multidimensional data, including equipment operating parameters and maintenance records. For instance, based on the correlation analysis of bearing temperature and vibration frequency, AI models can provide early warnings of mechanical wear risks, thereby offering a scientific basis for safety directors to organize preventive maintenance and effectively reducing the likelihood of safety accidents caused by equipment failure.

(2) Risk Processing Capability

Risk response capability focuses on “The timeliness and thoroughness of hazard rectification”. Its core lies in optimizing the rectification process through an AI-based hierarchical warning mechanism, thereby improving the efficiency and quality of risk treatment. The quantitative calculation formula is as follows:


$$\begin{array}{c} S_{\text{p}}=0.5\frac{N_{\text{rt}}}{N_{\text{r}}}+0.3\frac{N_{\text{1}}}{N_{\text{rt}}}+0.2\frac{\text{1}}{N_{\text{rn}}} \end{array}$$ (5)

Where, *S*_p_ represents the risk response capability score; *N*_rt_ represents the number of hazards rectified on schedule; *N*_r_ represents the total number of hazards requiring rectification; *N*_1_ represents the number of hazards that passed rectification in the first attempt; and *N*_m_ represents the number of rework instances during hazard rectification.

**Fig 4:**
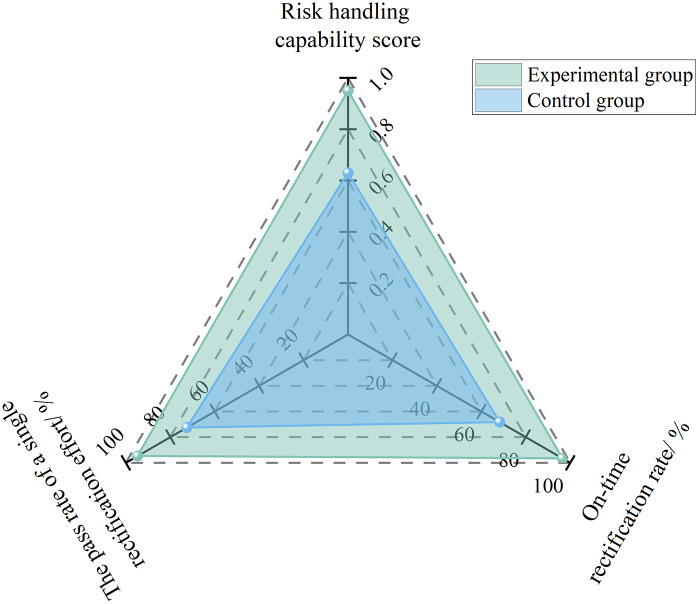
Experimental results for risk response capability under AI enablement.

To analyze the effect of AI models on improving safety directors’ risk response capability, 10 project safety directors were randomly selected from the experimental subjects described in Section 4.1.1 to participate in a six-month comparative experiment, with the on-schedule rectification rate and rectification qualification rate of hazards as the experimental focus. During the first three months, no AI-based hierarchical warning mechanism was adopted, whereas during the following three months, the AI-based hierarchical warning mechanism was applied. As shown in Fig 4, after the application of the AI-based hierarchical warning mechanism, the on-schedule rectification rate increased from 68.3% to 96.5%, the first-pass rectification qualification rate rose from 72.5% to 94.7%, the average number of rectification rework instances decreased from 3.2 times per month to 0.5 times per month, and the risk response capability score improved from 0.63 to 0.95, thereby achieving a rapid and efficient closed loop of risk treatment.

(3) Technical Application Capability

Technical application capability focuses on “The actual frequency and effectiveness of AI tool usage”. Its core lies in measuring safety directors’ mastery of AI technologies and their application outcomes. The quantitative calculation formula is as follows:


$$\begin{array}{c} S_{\text{t}}=0.4\frac{N_{\text{fr}}}{N_{\text{af}}}+0.3\frac{N_{\text{au}}}{N_{\text{pr}}}+0.3\frac{\text{1}}{T_{\text{ta}}} \end{array}$$ (6)

Where, *S*_t_ represents the technical application capability score; *N*_fr_ represents the number of AI applications; *N*_af_ represents the total number of work activities; *N*_au_ represents the number of hazards identified with AI assistance; *N*_pr_ represents the total number of hazards identified; and *T*_ta_ represents the average duration of AI application.

To analyze the effect of specialized AI technical training on improving safety directors’ technical application capability, 10 project safety directors were randomly selected from the experimental subjects described in Section 4.1.1 to participate in a six-month comparative experiment. During the first three months, no specialized learning related to AI technology was provided. At the beginning of the fourth month, specialized AI technical training was conducted. The training was designed to cover practical on-site application scenarios of AI tools, including hazard annotation and statistical analysis on intelligent monitoring platforms, parameter adjustment and result interpretation of risk prediction models, and other relevant tasks, so as to ensure that safety directors could deeply integrate AI technology with actual on-site safety management. For example, by using risk heat maps generated by AI tools, they could accurately identify high-risk work areas, optimize inspection routes and frequencies, and improve the precision and efficiency of on-site safety management.

**Fig 5:**
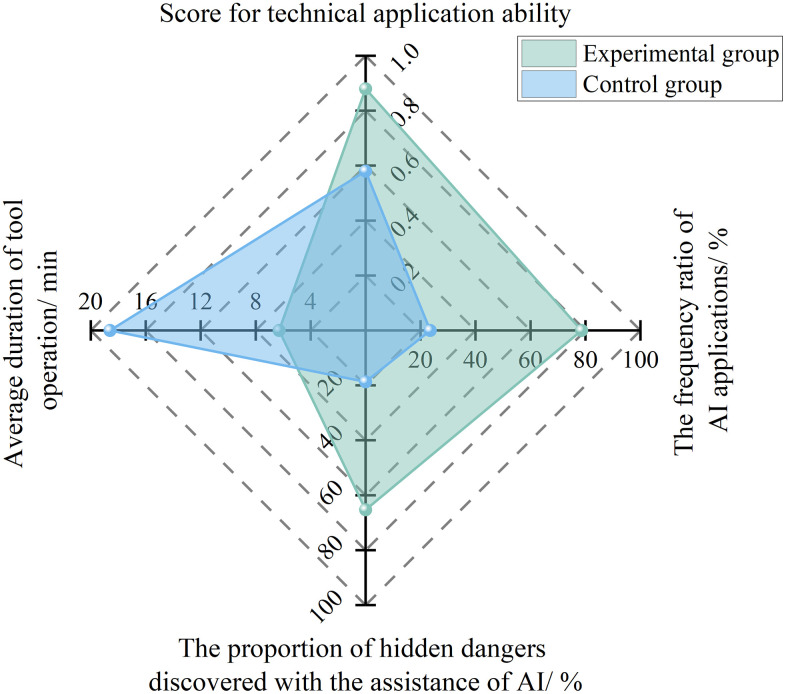
Experimental results for technical application capability under AI enablement.

As shown in Fig 5, after the specialized AI technical training, the proportion of AI application frequency increased from 23.5% to 78.6%, the proportion of hazards identified with AI assistance rose from 18.7% to 65.3%, the average tool operation time was reduced from 18.6 minutes to 6.3 minutes, and the technical application capability score improved from 0.58 to 0.88, realizing an upgrade in technical application capability from passive use to active optimization.

### 4.2 Enhancing collaborative management and strategic planning capability

Collaborative management capability and strategic planning capability constitute the core elements of EPC project safety directors’ competency, and are also key to their transformation from “task executors” to “strategic controllers”. Given that EPC projects involve multiple stakeholders, including design, construction, supervision, and subcontracting parties, differences in interests and management standards among these participants can easily lead to information barriers and responsibility shirking in cross-entity collaboration. At the same time, EPC projects span the entire life cycle, from planning and design to operation and maintenance, and the risk characteristics and safety requirements vary significantly across different stages, which places higher demands on safety directors’ strategic planning capability. Under the traditional management model, cross-party communication mainly relies on offline meetings and paper-based documents, facing prominent problems such as delayed information circulation and information asymmetry. In addition, strategic planning is often limited to personal experience and lacks data support from a full life-cycle perspective, making it difficult to achieve optimal resource allocation. Empowered by AI technologies such as intelligent decision support, precise collaboration platforms, and intelligent emergency response systems, safety directors can effectively unleash their strategic thinking and coordination potential, thereby promoting collaborative management and strategic planning toward a more precise, efficient, and systematic mode.

#### 4.2.1 Precision collaboration platform.

Collaborative management capability focuses on the “task completion rate and communication efficiency among participating parties”. Its core lies in breaking down information barriers and achieving efficient cross-party collaboration through an AI-powered precise collaborative management platform. The quantitative calculation formula is as follows:


$$\begin{array}{c} F_{\text{s}}=0.4\frac{R_{\text{cs}}}{R_{\text{at}}}+0.3\frac{R_{\text{nc}}}{R_{\text{at}}}+0.3\frac{\text{1}}{T_{\text{ct}}} \end{array}$$ (7)

Where, *F*_s_ is the collaborative management capability score, *R*_cs_ is the number of cross-party collaborative tasks completed on schedule, *R*_at_ is the total number of collaborative tasks, *R*_nc_ is the number of conflict-free collaborative tasks, and *T*_ct_ is the average communication duration.

To analyze the effectiveness of the precise collaborative management platform in improving safety directors’ collaborative management capability, an empirical study was conducted based on the Hangzhou Grand Convention and Exhibition Center Project, the Qingyuan Vocational Education Second Road Engineering Project, and the Shenzhen Baguang Comprehensive Sports Center Project. After the platform was applied, project safety directors were able to realize real-time transmission and traceability of information such as safety management regulations and hazard rectification requirements through a unified information-sharing module. All participating parties could access relevant information according to their authorization levels, thereby avoiding information asymmetry. Through the collaborative task management module, collaborative tasks could be created with clearly defined responsible entities and completion deadlines. The system automatically tracked task progress and issued reminders for overdue tasks, thus forming a closed-loop management mechanism for collaborative tasks.

**Fig 6:**
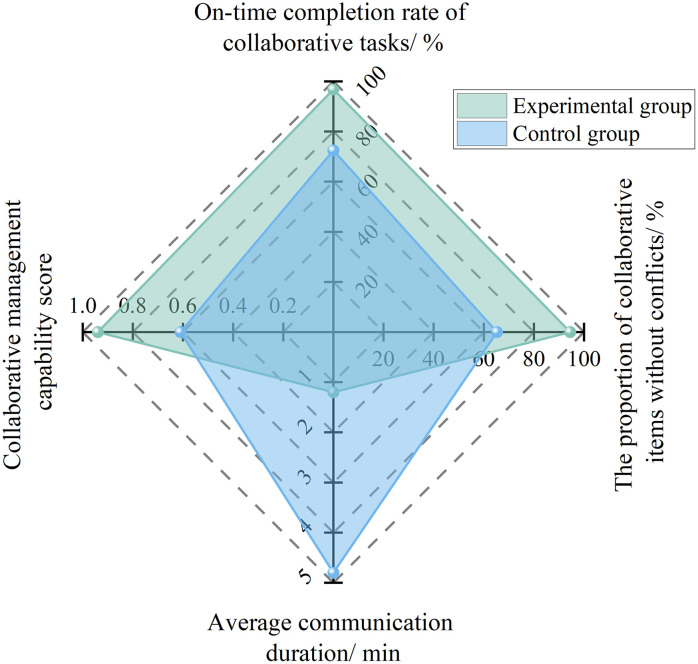
Experimental results of collaborative management capability under AI empowerment.

The empirical results are shown in Fig 6. The on-time completion rate of collaborative tasks increased from 72.5% to 96.8%, the average communication duration was reduced from 4.8 h to 1.2 h, the proportion of conflict-free collaborative items increased from 65.3% to 94.5%, and the collaborative management capability score rose from 0.61 to 0.94, representing an improvement of 54.1%.

In addition, the trust mechanism established through blockchain technology ensures the immutability and traceability of collaborative information, significantly enhancing the “authority recognition” of safety directors in cross-party coordination. This further strengthens their central role in overall coordination and effectively addresses problems such as responsibility shirking and process bottlenecks among participating parties.

#### 4.2.2 Intelligent decision support system.

Strategic planning capability focuses on the “implementation rate of safety planning and input–output effectiveness.” Its core lies in leveraging an AI-based intelligent decision-support system to integrate full-life-cycle data and industry experience, thereby supporting safety directors in formulating scientific and reasonable safety plans. The quantitative calculation formula is as follows:


$$\begin{array}{c} F_{\text{t}}=0.6\frac{R_{\text{pc}}}{R_{\text{ap}}}+0.4\frac{R_{\text{pd}}}{R_{\text{ad}}} \end{array}$$ (8)

Where, *F*_t_ is the strategic planning capability score, *R*_pc_ is the number of plans successfully implemented, *R*_ap_ is the total number of plans, *R*_pd_ is the number of hazards covered throughout the full life cycle, and *R*_ad_ is the total number of hazards.

To analyze the effect of the intelligent decision-support system on improving safety directors’ strategic planning capability, an empirical study was conducted based on the Hangzhou Grand Convention and Exhibition Center Project, the Qingyuan Vocational Education Second Road Engineering Project, and the Shenzhen Baguang Comprehensive Sports Center Project. After the system was applied, safety directors were able to use it to integrate geotechnical investigation data, construction schedule data, historical hazard data, and other relevant information, and to formulate full-life-cycle safety plans that clearly defined the key risks and control measures at each stage.

**Fig 7:**
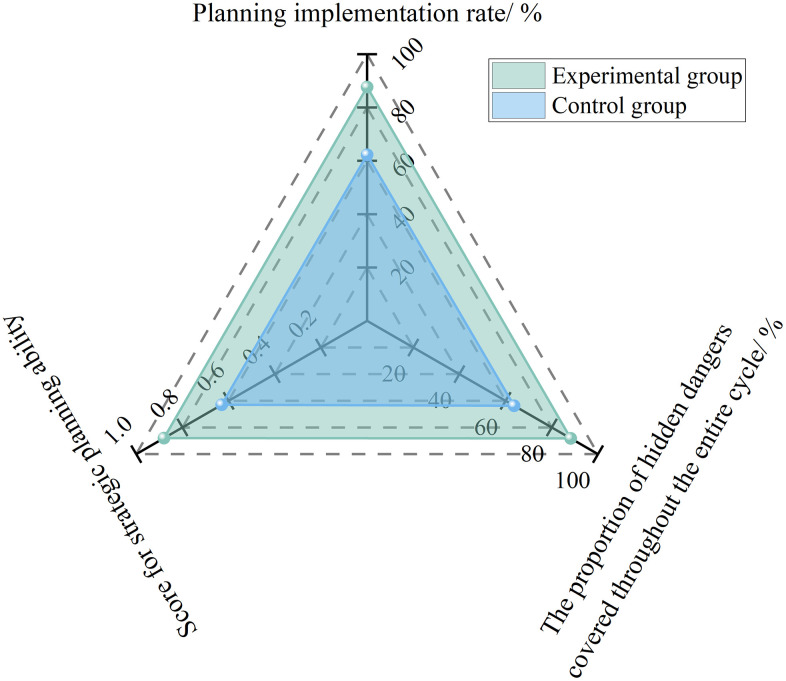
Experimental results of strategic planning capability under AI empowerment.

The empirical results are shown in Fig 7. The planning implementation rate increased from 62.3% to 87.7%, the proportion of hazards covered throughout the full life cycle rose from 63.7% to 88.2%, and the number of hazards reduced per RMB 10,000 of safety investment increased from 2.3 to 6.8. The strategic planning capability score improved from 0.63 to 0.88, representing an increase of 39.7%.

In summary, the integration of digital twin technology further enhances the visualization and operability of strategic planning. By using virtual models to simulate the implementation effects of different planning schemes—such as evaluating the stability of various deep foundation pit support designs under heavy rainfall conditions—safety directors can identify the optimal solution, avoid a disconnect between planning and on-site realities, and improve the scientific soundness and feasibility of safety planning.

### 4.3 Promoting continuous learning and regulatory policy tracking

Continuous learning ability and the capacity to keep pace with laws, regulations, and policies are essential safeguards for the sustained improvement of safety directors’ competencies and are also core qualities that enable them to adapt to the digital transformation of the industry. As the digital and intelligent transformation of the engineering construction industry continues to accelerate, the application of AI technologies in the field of safety management is deepening, and new technological tools, management concepts, and regulatory standards are constantly emerging, thereby placing higher demands on safety directors’ continuous learning ability. Meanwhile, with the continuous improvement of laws and regulations such as the Work Safety Law and the Regulations on the Administration of Work Safety in Construction Projects, EPC projects are imposing increasingly stringent requirements on safety directors’ ability to keep up with relevant laws, regulations, and policies.

Under the traditional model, problems such as insufficiently targeted training, difficulties in knowledge accumulation, and delays in regulatory updates have constrained the continuous improvement of these two capabilities. By contrast, through systematic empowerment based on a hierarchical and categorized training system, efficient knowledge management mechanisms, and a continuous culture cultivation model, AI has established a closed-loop learning system integrating “training–learning–innovation”, thereby providing support for the long-term enhancement of safety directors’ competencies.

#### 4.3.1 Continuous learning capability.

Continuous learning ability focuses on the “training compliance rate and the effectiveness of knowledge application”. Its core lies in using an AI-based hierarchical and categorized training system to accurately match the competency needs of safety directors at different levels, thereby improving training outcomes and the conversion rate of knowledge application. The quantitative calculation formula is as follows:


$$\begin{array}{c} L_{\text{cl}}=0.4\frac{T_{\text{qn}}}{T_{\text{at}}}+0.4\frac{T_{\text{ka}}}{T_{\text{at}}}+0.2Q_{\text{drr}} \end{array}$$ (9)

Where, *L*_cl_ is the continuous learning ability score, *T*_at_ represents the total number of training sessions, *T*_qn_ refers to the number of training sessions successfully completed, *T*_ka_ indicates the number of successful applications of the learned content, and *Q*_drr_ is the rate of hazard reduction after training.

To analyze the effect of the AI-based hierarchical and categorized training system on improving the continuous learning ability of safety directors, a six-month comparative experiment was conducted with 20 project safety directors selected in Section 4.1.1 as the research subjects. Based on their competency evaluation results, the 10 participants in the experimental group used the AI-based hierarchical and categorized training system to accurately identify competency gaps at different levels and develop targeted training plans, whereas the 10 participants in the control group followed the company’s general training program. During the experiment, the learning content, training outcomes, and work performance of all participants were recorded.

**Fig 8:**
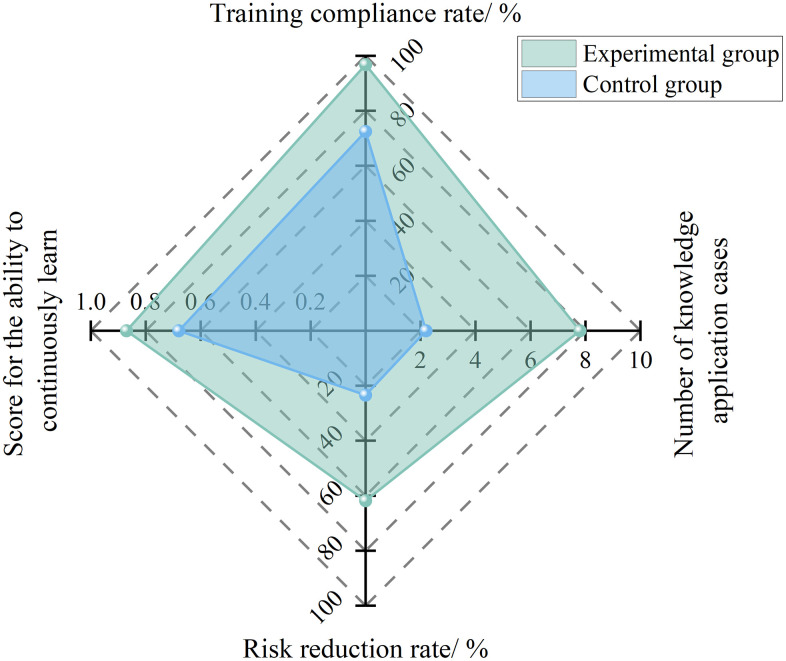
Experimental results of continuous learning capability under AI empowerment.

The experimental results are shown in Fig 8. After the adoption of the AI-based hierarchical and categorized training system, the training compliance rate increased from 72.5% to 96.8%, the number of knowledge application cases rose from an average of 2.2 to 7.8 cases per person per half-year, and the six-month hazard reduction rate improved from 23.4% to 61.8%. Accordingly, the average score for continuous learning ability increased from 0.68 to 0.87, demonstrating a precise and targeted improvement in this capability.

In summary, the personalized training needs matching model uses the cosine similarity algorithm to match the competency demand vectors of safety directors with training content vectors, achieving a matching accuracy of 89.3%. This effectively avoids the inefficiency of “one-size-fits-all” training and ensures a high degree of alignment between the training content and the actual needs of safety directors.

#### 4.3.2 Regulatory policy tracking capability.

Regulatory and policy tracking capability focuses on “the timeliness of regulatory and policy acquisition, the accuracy of interpretation, and the adaptability of implementation”. Its core lies in using AI technology to monitor policy updates in real time, provide concrete interpretations of policy provisions, clarify the correspondence between policy requirements and on-site safety management, and offer case references and implementation guidance. This helps safety directors quickly capture updates in industry regulations, policies, and standards, accurately interpret key requirements, and efficiently adapt them to project safety management practice. The quantitative calculation formula is as follows:


$$\begin{array}{c} L_{\text{pr}}=0.6\frac{T_{\text{in}}}{T_{\text{un}}}+0.4\frac{T_{\text{an}}}{T_{\text{in}}} \end{array}$$ (10)

Where, *L*_pr_ represents the score of regulatory and policy tracking capability, *T*_un_ is the number of relevant regulatory and policy updates within a specified period, *T*_in_ is the number of newly updated policies and regulations studied within the specified period, and *T*_an_ is the number of applications of the latest policies and regulations.

To analyze the effect of AI technology on improving the regulatory and policy tracking capability of safety directors, a six-month comparative experiment was conducted using the 20 project safety directors selected in Section 4.1.1 as the research subjects. The experimental group, consisting of 10 participants, tracked, interpreted, and applied the latest regulations and policies with AI assistance, while the control group, also consisting of 10 participants, did not use any AI-assisted tools.

**Fig 9:**
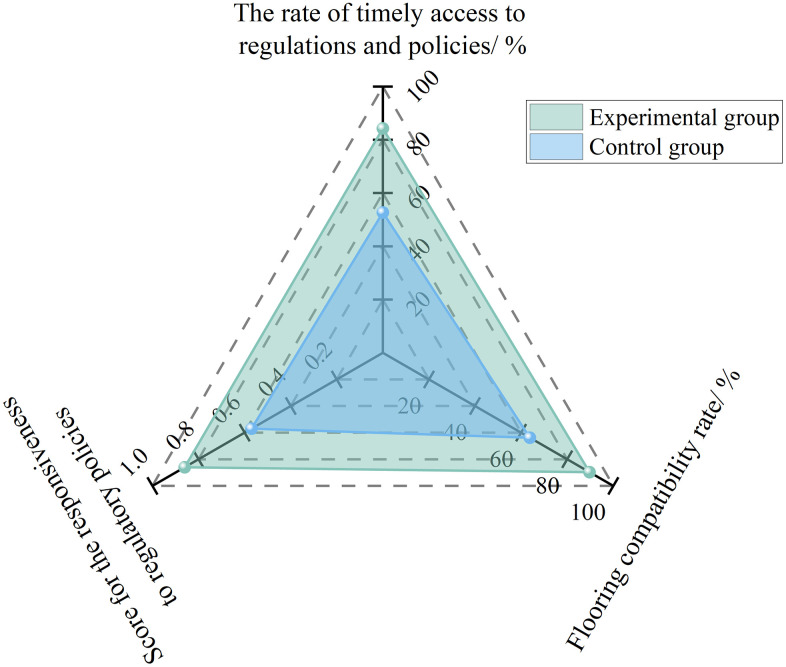
Experimental results of regulatory and policy tracking capability under AI empowerment.

The experimental results are shown in Fig 9. After adopting AI-assisted tools, the timely acquisition rate of regulations and policies increased from 52.7% to 84.3%, the implementation adaptability rate rose from 63.8% to 89.7%, and the score of regulatory and policy tracking capability improved from 0.57 to 0.86, representing an increase of 50.9%.

In summary, AI technology has demonstrated significant effectiveness in enhancing the regulatory and policy tracking capability of safety directors. By enabling real-time monitoring, accurate policy interpretation, and providing implementation-oriented adaptation solutions, it effectively addresses key challenges such as the timeliness of policy acquisition, the accuracy of interpretation, and the adaptability of implementation.

### 4.4 Comprehensive quantitative evaluation of safety director competency

To further quantify the overall impact of AI empowerment on the competency structure of safety directors and explore the coupling and synergistic effects among different competency dimensions, this section integrates the independent empirical analyses of sub-capabilities such as risk control, collaborative planning, and continuous learning presented above to construct a comprehensive evaluation model for safety directors’ competency.

#### 4.4.1 Construction of the comprehensive competency index.

To achieve a unified evaluation of heterogeneous indicators, this study maps and aggregates the six categories of quantitative indicators defined above into three core dimensions based on the “sensing–seizing–reconfiguring” framework of dynamic capability theory. Combined with Equation (2), a Comprehensive Competency Index (*CCI*) is constructed. The calculation formula is as follows:


$$\begin{array}{c} CCI=\omega_{\text{1}}S_{\text{sen}}+\omega_{\text{2}}S_{\text{sei}}+\omega_{\text{3}}S_{\text{tra}} \end{array}$$ (11)

$$\begin{array}{c} \left\{ \begin{array}{c} @l@S_{\text{sen}}=\frac{\text{1}}{\text{2}}\left(C_{\text{mc}}+S_{\text{t}}\right)\\ S_{\text{sei}}=\frac{\text{1}}{\text{2}}\left(F_{\text{s}}+F_{\text{t}}\right)\\ S_{\text{tra}}=\frac{\text{1}}{\text{2}}\left(L_{\text{cl}}+L_{\text{pr}}\right) \end{array}\right. \end{array}$$ (12)

$$\begin{array}{c} C_{\text{mc}}=\frac{\text{1}}{\text{3}}\left(C_{\text{r}}+S_{\text{w}}+S_{\text{p}}\right) \end{array}$$ (13)

Where, *S*_sen_, *S*_sei_, and *S*_tra_ represent the scores for sensing capability, seizing capability, and reconfiguring capability, respectively, while *ω*_1_, *ω*_2_, and *ω*_3_ is the corresponding weight coefficients. *C*_mc_ is the score for risk control capability, *S*_t_ the score for technology application capability, *F*_s_ the score for collaborative management capability, *F*_t_ the score for strategic planning capability, *L*_cl_ the score for continuous learning capability, and *L*_pr_ the score for regulatory and policy tracking capability. In addition, *C*_r_, *S*_w_, and *S*_p_ is the scores for risk identification capability, risk anticipation capability, and risk response capability, respectively.

To ensure that the *CCI* can scientifically reflect the characteristics of competency improvement of safety directors under AI empowerment, this study adopts the AHP to determine the weight coefficients*ω*_1_, *ω*_2_, and *ω*_3_ for *S*_sen_, *S*_sei_, and *S*_tra_. The specific method is as follows.

First, a review panel consisting of 12 experts was invited, all of whom held either doctoral degrees or senior professional technical titles and had more than 10 years of experience in the field. Based on the Saaty 1–9 scale method, the three core dimensions were compared pairwise according to the decision-making characteristics of safety directors in complex risk environments. The judgment matrix was then constructed using the geometric mean of the experts’ ratings, as follows:


$$\begin{array}{c} A=\left(\begin{array}{ccc} @ccc@1 & 0.88 & 1.4\\ 1.14 & 1 & 1.6\\ 0.71 & 0.63 & 1 \end{array}\right) \end{array}$$ (14)

This matrix reflects the expert panel’s assessment of the composition of safety directors’ competency: in an operational environment where AI technologies are deeply embedded, collaborative management and strategic planning capabilities are considered the most important, followed by front-end risk control and technology application capabilities. Although continuous learning and regulatory/policy tracking capabilities are recognized as valuable for improvement, they carry relatively lower weights in dynamic response contexts.

Second, the eigenvector method was employed to solve the judgment matrix. The resulting eigenvector was then normalized, and the weight coefficients of the three core dimensions were finally determined as *ω*_1_ = 0.35, *ω*_2_ = 0.40 and *ω*_3_ = 0.25.

Finally, to verify the logical consistency of the experts’ judgments and avoid contradictions such as “A is more important than B, B is more important than C, but C is more important than A”, this study introduces the Consistency Ratio (*CR*) for testing. The calculation method is as follows:


$$\begin{array}{c} CI=\frac{\lambda_{max}-n}{n-1} \end{array}$$ (15)

$$\begin{array}{c} CR=\frac{CI}{RI} \end{array}$$ (16)

Where, *CI* is the Consistency Index, *λ*_max_ is the maximum eigenvalue (in this study, *λ*_max_ = 3), *n* is the number of indicators involved in the comparison (in this study, *n* = 3), *CR* is the Consistency Ratio, and *RI* is the Random Consistency Index (when n=3, RI=0.58).

The calculation results show that *CI* = 0.0005 and *CR* = 0.00086. Since *CR* is far below 0.1, the judgment matrix demonstrates excellent consistency, indicating that the allocation of weight coefficients is scientifically sound and can be used for the subsequent quantitative evaluation of the Comprehensive Competency Index.

#### 4.4.2 Overall efficacy analysis.

**Fig 10:**
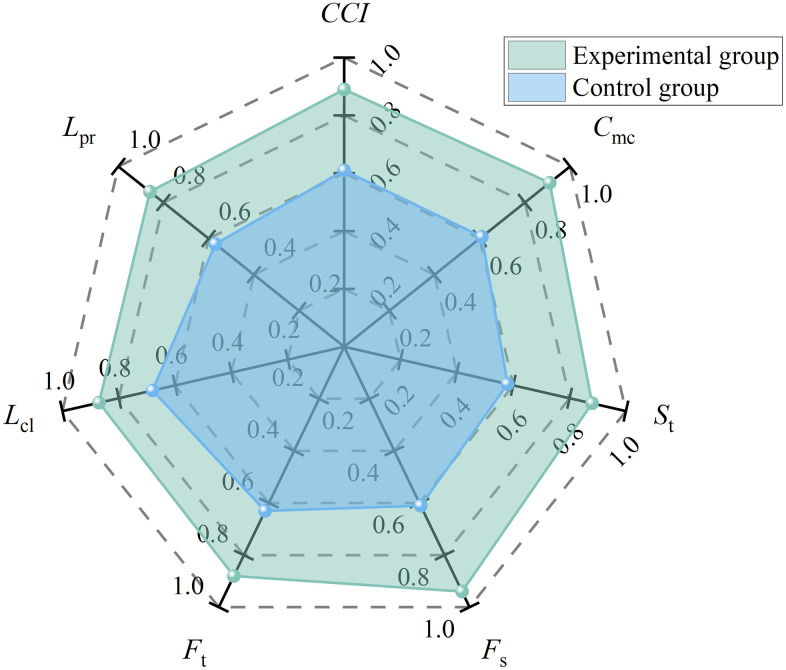
*CCI* calculation results.

Based on the empirical evaluation data from the experimental group (AI-empowered mode) and the control group (traditional management mode), the relevant indicators were incorporated into the constructed *CCI* model for quantitative comparison. SPSS software was used to conduct an independent samples t-test and effect size analysis on the experimental data. The results show that the *CCI* of the experimental group was significantly higher than that of the control group, with mean values of 0.89 (*SD* = 0.04) and 0.61 (*SD* = 0.05), respectively. The independent samples t-test yielded *t*_(18)_ = 13.85，*p* < 0.001, indicating an extremely significant difference. The 95% confidence interval was [0.23, 0.33], which did not include 0. Meanwhile, Cohen’s d was 6.19, far exceeding the threshold of 0.8 for a large effect. The detailed results are presented in Fig 10 and Table 1.

**Table 1:** Calculation results of *CCI* parameters.

Group	*C*_mc_	*S*_t_	*F*_s_	*F*_t_	*L*_cl_	*L*_pr_	*CCI*
Experimental Group	0.91	0.88	0.94	0.88	0.87	0.86	0.89
Control Group	0.61	0.58	0.61	0.63	0.68	0.57	0.61

These findings were obtained under the strict assumption that initial differences in background knowledge and technical capability between the two groups were controlled. This indicates that the 45.9% increase did not result from the experimental group members having superior engineering experience or a natural sensitivity to emerging technologies, but rather accurately reflects the substantive expansion of individual cognitive boundaries enabled by the AI-empowered mode. Under the traditional management mode, even safety directors with extensive background knowledge remain constrained by physiological energy and the bandwidth limitations of human information processing. The introduction of innovative AI technologies bridges the gap between the upper limits of human experience and the vast volume of complex on-site data. Therefore, when engineering personnel begin with comparable initial capabilities, those who are able to integrate innovative technological tools into their daily decision-making processes at an earlier stage can gain a clear performance advantage in the safety management of dynamic and complex EPC projects.

In summary, the integration of AI technology is not merely a localized refinement of traditional management tools; rather, through the digital reconstruction of underlying operational logic, it drives a qualitative transformation in the competency of safety directors from experience-driven to data-driven performance. AI empowerment breaks through the bottlenecks of individual effectiveness inherent in traditional management modes, enabling safety directors to maintain superior decision-making performance in highly complex and dynamic working environments.

### 4.5 Hierarchical improvement strategies for project safety director competency

Based on the above comprehensive quantitative evaluation results, and in view of the highly dynamic and strongly coupled characteristics of EPC projects, this study proposes the following four AI-empowered strategies for improving the competency of project safety directors:

1. Data-driven proactive risk prediction strategy: To address the limitations of restricted managerial vision and high omission rates under the traditional mode, safety directors should take the lead in establishing a project-level Internet of Things system based on “multi-source data fusion”. Through the round-the-clock deployment of computer vision and sensor networks, the focus of safety management can be shifted forward from “hazard inspection” to “real-time monitoring of risk factors”. AI models can then be used to identify weak-signal risks, thereby moving the line of defense in risk control to an earlier stage.
2. Cross-domain integrated intelligent collaborative governance strategy: To overcome information barriers among multiple stakeholders involved in design, procurement, and construction in EPC projects, safety directors should rely on precision collaborative management platforms and BIM/DW technologies to formulate standardized data-sharing protocols across participating parties. With the support of AI-assisted intelligent decision-making systems, hazard rectification instructions can be automatically distributed and tracked in a closed-loop manner, reducing communication costs and reshaping an efficient cross-organizational collaboration mechanism.
3. Dynamically matched personalized continuous empowerment strategy: The traditional “one-size-fits-all” safety training model should be abandoned. AI algorithms can be used to analyze the historical decision-making data and competency deficiencies of safety directors and project management teams. By matching demand vectors with training-content vectors through cosine similarity, relevant regulatory updates, accident cases, and frontier technologies can be accurately recommended, thereby transforming the learning process from passive reception to proactive, needs-based learning.
4. Human–AI trust-based agile decision-making and ethical governance strategy: When introducing AI systems, enterprises should clearly define the boundaries between human and machine tasks. Safety directors should not rely entirely on algorithms; instead, AI should be positioned as a “cognitive prosthesis” and an “efficiency multiplier”. While AI automates the processing of massive volumes of routine data, safety directors must retain the central leading role in complex situational judgment, major risk trade-offs, and safety ethics governance, thereby establishing a healthy human–AI trust mechanism.

## 5. Conclusion and outlook

### 5.1 Conclusion

This study systematically investigates the empowerment pathways and empirical effects of AI technology on the competency of safety directors in EPC projects by constructing a dynamic capability framework of “perception–capture–reconfiguration”. The main conclusions are as follows:

(1) AI technology drives a leap in competency level. The empirical results show that, under the AI-empowered mode, the overall CCI of safety directors increased from 0.61 in the control group to 0.89, representing an increase of 45.9%. In particular, for information-intensive tasks, both the accuracy and timeliness of regulatory and policy matching improved substantially. This indicates that AI technology is not merely an optimization of management tools, but also a reshaping of the competency structure through cognitive enhancement.
(2) The management paradigm undergoes a profound transformation from “experience-driven” to “data-driven”. The study finds that AI technology effectively compensates for the limitations of individual cognitive bandwidth by digitally reconstructing the underlying management logic. Safety directors are thus able to capture weak-signal risks in highly complex environments in real time, enabling a qualitative shift in risk identification from post-event response to pre-event prediction, and significantly enhancing the scientific basis and precision of decision-making.
(3) The evaluation model demonstrates a high degree of scientific rigor and logical consistency. The consistency ratio test (CR=0.00086≪0.1) verifies that the weight allocation of the competency evaluation indicators constructed in this study is rigorous and reasonable. This model not only objectively reflects the actual effects of AI empowerment, but also provides a replicable theoretical framework for the quantitative evaluation of safety management talent in the EPC industry.
(4) Targeted hierarchical improvement strategies are proposed. First, a proactive risk prediction strategy centered on all-weather IoT-based perception is suggested. Second, a cross-domain collaborative governance strategy based on digital twin technology and collaborative platforms is proposed. Third, a personalized continuous empowerment strategy driven by AI similarity algorithms is advanced. Fourth, a human–AI trust governance strategy is recommended, emphasizing clear task boundaries between humans and machines while preserving human ethical leadership. These strategies provide practical guidance for enterprise talent development during digital and intelligent transformation.

### 5.2 Outlook

Although this study has achieved phased progress in both theoretical construction and empirical analysis, several aspects still require further exploration due to objective constraints:

(1) Limitations in sample size and generalizability. Restricted by the scarcity of senior safety directors in large EPC projects and the practical difficulty of conducting a long-cycle (six-month) in-depth intervention experiment, this study included only 20 participants. Although this satisfies the basic statistical requirements of a quasi-experimental design, the relatively small sample size may limit the broader generalizability of the findings to some extent. Future research should expand the sample size and carry out multi-center, large-sample validation across different regions and industries, such as municipal engineering and building construction.
(2) Psychological mechanisms of technology adoption and behavioral evolution. Subsequent studies may introduce the TAM and the Unified Theory of Acceptance and Use of Technology to more deeply analyze the dynamic evolution of safety directors’ perceived usefulness, perceived ease of use, and willingness to adopt AI tools. This would help reveal the underlying psychological variables that influence the effectiveness of technology application.
(3) Insufficient quantification of practical implementation barriers and organizational resistance. This paper mainly verifies the empowering potential of AI technology, but its implementation in engineering practice still faces multiple challenges. First, implementation costs remain high, as the deployment of software, hardware, and network communication infrastructure may constrain practical application at the project level. Second, data silos and privacy barriers coexist; fragmented data across participating stakeholders may cause algorithms to fail due to the lack of high-quality training data. Third, organizational resistance remains significant, as some senior managers may distrust the “black box” nature of algorithms or experience professional anxiety due to concerns about technological substitution. Future research should incorporate advanced evolutionary perspectives within technology acceptance theory to systematically examine how these practical barriers weaken the effects of AI empowerment and to explore corresponding pathways for organizational change.

## References

[1] Jiang WP, Tang SQ. The cooperation establishment mechanism of EPC project consortium in context of China: form the perspective of trust. Sustainability. 2023;15(2). doi: 10.3390/su15021266

[2] Ghasemi M, Nejad MG, Alsaadi N, Abdel-Jaber M, Ab Yajid MS, Habib M. Performance measurement and lead-time reduction in EPC project-based organizations: a mathematical modeling approach. Math Probl Eng. 2022. doi: 10.1155/2022/5767356

[3] Wu YJ, He XM, Cui TY, Wu MZ. Decision-making evaluation and optimization strategies for construction EPC project developers utilizing BIM technology. Adv Civil Eng. 2024. doi: 10.1155/2024/6694580

[4] Zhang RP, Pirzadeh P, Lingard H, Nevin S. Safety climate as a relative concept: exploring variability and change in a dynamic construction project environment. Eng Const Arch Manag. 2018;25(3):298–316. doi: 10.1108/ECAM-09-2016-0207

[5] Koc K, Ekmekcioğlu Ö, Gurgun AP. Developing a national data-driven construction safety management framework with interpretable fatal accident prediction. J Constr Eng Manage. 2023;149(4):04023010. doi: 10.1061/JCEMD4.COENG-12848

[6] Chen F, Wang H, Xu G, Ji H, Ding S, Wei Y. Data-driven safety enhancing strategies for risk networks in construction engineering. Reliab Eng Syst Saf. 2020;197:106806. doi: 10.1016/j.ress.2020.106806

[7] Chang S-H, Chen D-F, Wu T-C. Developing a competency model for safety professionals: correlations between competency and safety functions. J Safety Res. 2012;43(5–6):339–50. doi: 10.1016/j.jsr.2012.10.009 23206506

[8] Khawam AA, Bostain NS. Project manager’s role in safety performance of Saudi construction. Int J Manag Proj Business. 2019;12(4):938–60. doi: 10.1108/ijmpb-04-2018-0087

[9] Zhao Z, Gao Y, Hu X, Zhou Y, Zhao L, Qin G, et al. Integrating BIM and IoT for smart bridge management. In: IOP Conf. Ser.: Earth Environ. Sci, 2019. 022034. doi: 10.1088/1755-1315/371/2/022034

[10] Hu Z, Zhang J, Deng Z. Construction process simulation and safety analysis based on building information model and 4D technology. Tinshhua Sci Technol. 2008;13(S1):266–72. doi: 10.1016/s1007-0214(08)70160-3

[11] Park J, Kang D. Artificial intelligence and smart technologies in safety management: a comprehensive analysis across multiple industries. Appl Sci. 2024;14(24):11934. doi: 10.3390/app142411934

[12] Dobrucali E, Demirkesen S, Sadikoglu E, Zhang C, Damci A. Investigating the impact of emerging technologies on construction safety performance. ECAM. 2022;31(3):1322–47. doi: 10.1108/ecam-07-2022-0668

[13] Wang Y. Research on the application of smart construction site in safety management of engineering construction. Appl Comp Eng. 2015;187:65–74. doi: 10.54254/2755-2721/2025.GL27811

[14] Kulinan AS, Park M, Aung PPW, Cha G, Park S. Advancing construction site workforce safety monitoring through BIM and computer vision integration. Autom Constr. 2024;158:105227. doi: 10.1016/j.autcon.2023.105227

[15] Xu Q, Liu L, Zhang F, Ma X, Sun K, Cui F. An intelligent recognition method of factory personnel behavior based on deep learning. Digital Signal Process. 2025;156:104834. doi: 10.1016/j.dsp.2024.104834

[16] Wu S, Hou L, Zhang G, Chen H. Real-time mixed reality-based visual warning for construction workforce safety. Autom Constr. 2022;139:104252. doi: 10.1016/j.autcon.2022.104252

[17] Wang J, Fan Y, Pan X, Sun J, Zhang L. Multi-source information fusion for dynamic safety risk prediction of aerial building machine using spatial–temporal multi-graph convolution network. Advances in Engineering Information. 2025;65:103261. doi: 10.1016/j.aei.2025.103261

[18] Sun H, Zhu M, Dai Y, Liu X, Li X. Dynamic risk early warning system for tunnel construction based on two-dimensional cloud model. Expert Syst Appl. 2024;255:124799. doi: 10.1016/j.eswa.2024.124799

[19] Huo X, Yin Y, Jiao L, Zhang Y. A data-driven and knowledge graph-based analysis of the risk hazard coupling mechanism in subway construction accidents. Reliabil Eng System Safety. 2024;250:110254. doi: 10.1016/j.ress.2024.110254

[20] Zhang X, Tian D, Ren Q, Li M, Shen Y, Han S. A hybrid deep semantic mining method considering fuzzy expressions for the automatic recognition of construction safety hazard information. Adv Eng Inform. 2024;61:102507. doi: 10.1016/j.aei.2024.102507

[21] Moe SJS, Rizwan A, Khan AN, Xu R, Kim DH. Safety monitoring digital twin-based centralized model consolidation mechanism using dynamic node selection for multi-worker safety prediction. Eng Appl Artif Intell. 2025;161:112186. doi: 10.1016/j.engappai.2025.112186

[22] Shi G, Liu Z, Lu D, Wang Z, Jiao Z, Ji C, et al. Construction error control method of large-span spatial structures based on digital twin. J Build Eng. 2024;98:111311. doi: 10.1016/j.jobe.2024.111311

[23] Han Y, Chen M, Li N, Ji M, Wang X. Digital twin in construction safety management: Recent advances, challenges, and future directions from 4M1E perspective. Saf Sci. 2025;192:107006. doi: 10.1016/j.ssci.2025.107006

[24] Davis FD, Bagozzi RP, Warshaw PR. User acceptance of computer technology: a comparison of two theoretical models. Manag Sci. 1989;35(8):982–1003. doi: 10.1287/mnsc.35.8.982

[25] Zhu K, Qian Y, Guo N. Public acceptance of on-street charging for electric vehicles in China: an extension of the UTAUT model. Transport Res Part F: Traffic Psychol Behav. 2026;118:103504. doi: 10.1016/j.trf.2025.103504

[26] Zhao C, Liu R, Wan Q, Xu X, He J. A human–machine collaboration driven criteria system intelligent construction method for sustainable offshore wind farm site selection. Comput Ind Eng. 2026;211:111611. doi: 10.1016/j.cie.2025.111611

[27] Wang L, Zhang M, Li H, Hu Y, Ma J, Umer W, et al. Modeling trust in human–robot collaborative construction: an improved cloud Bayesian network. Expert Syst Appl. 2026;298:129928. doi: 10.1016/j.eswa.2025.129928

[28] Teece DJ. Explicating dynamic capabilities: the nature and microfoundations of (sustainable) enterprise performance. Strat Manag J. 2007;28(13):1319–50. doi: 10.1002/smj.640

[29] Zhao P, Luo Z, Yang G, Cai W, Li L, Qin Y. Research on artificial intelligence algorithm system for medical institutions based on construction engineering and e-commerce. Highlights Sci Eng Technol. 2024;87. doi: 10.54097/kfgjw731

[30] Flemisch F, Abbink DA, Itoh M, Pacaux-Lemoine M-P, Weßel G. Joining the blunt and the pointy end of the spear: towards a common framework of joint action, human–machine cooperation, cooperative guidance and control, shared, traded and supervisory control. Cogn Tech Work. 2019;21(4):555–68. doi: 10.1007/s10111-019-00576-1

[31] Maciejewski ML. Quasi-experimental design. Biostatistics & Epidemiology. 2018;4(1):38–47. doi: 10.1080/24709360.2018.1477468

[32] S. T. The analytic hierarchy process. New York: MeGraw-HillInc; 1980.

[33] Hu J, Tang S, Yang M. Risk evaluation of BIM technology application in EPC projects. Eng Econ. 2021;31(4):68–73. doi: 10.19298/j.cnki.1672-2442.202104068

